# De novo biosynthesis of *C*-arabinosylated flavones by utilization of *indica* rice *C*-glycosyltransferases

**DOI:** 10.1186/s40643-021-00404-3

**Published:** 2021-06-12

**Authors:** Zhuo Chen, Yuwei Sun, Guangyi Wang, Ying Zhang, Qian Zhang, Yulian Zhang, Jianhua Li, Yong Wang

**Affiliations:** 1 CAS-Key Laboratory of Synthetic Biology, CAS Center for Excellence in Molecular Plant Sciences, Institute of Plant Physiology and Ecology, Chinese Academy of Sciences, Shanghai, 200032 China; 2 University of Chinese Academy of Sciences, Beijing, 100039 China

**Keywords:** De novo biosynthesis, *C*-Arabinoside flavone, *C*-Glycosyltransferase, Rice

## Abstract

Flavone *C*-arabinosides/xylosides are plant-originated glycoconjugates with various bioactivities. However, the potential utility of these molecules is hindered by their low abundance in nature. Engineering biosynthesis pathway in heterologous bacterial chassis provides a sustainable source of these *C*-glycosides. We previously reported bifunctional *C*-glucosyl/*C*-arabinosyltransferases in *Oryza sativa* japonica and *O. sativa* indica, which influence the *C*-glycoside spectrum in different rice varieties. In this study, we proved the *C*-arabinosyl-transferring activity of rice *C*-glycosyltransferases (CGTs) on the mono-*C*-glucoside substrate nothofagin, followed by taking advantage of specific CGTs and introducing heterologous UDP-pentose supply, to realize the production of eight different *C*-arabinosides/xylosides in recombinant *E. coli*. Fed-batch fermentation and precursor supplement maximized the titer of rice-originated *C*-arabinosides to 20–110 mg/L in an *E. coli* chassis. The optimized final titer of schaftoside and apigenin di-*C*-arabinoside reached 19.87 and 113.16 mg/L, respectively. We demonstrate here the success of de novo bio-production of *C*-arabinosylated and *C*-xylosylated flavones by heterologous pathway reconstitution. These results lay a foundation for further optimal manufacture of complex flavonoid compounds in microbial cell factories.

**Supplementary Information:**

The online version contains supplementary material available at 10.1186/s40643-021-00404-3.

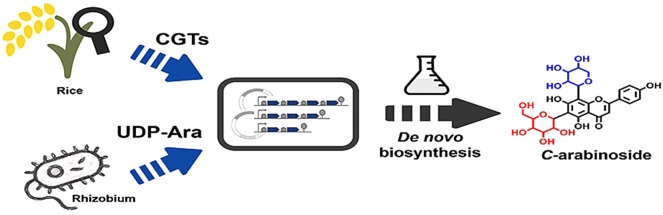

## Introduction

Flavone *C*-arabinosides are one of the less common classes of flavonoid glycosides occurring in nature. Despite of their rarity, many flavones bearing *C*-arabinosyls have been reported to show intriguing physiological activities. For example, schaftoside (apigenin 6-*C*-glucosyl-8-*C*-arabinoside, Sch) is present as a major component in the Chinese herb *Desmodium styracifolium*, possessing diverse bioactivities including antioxidant, anti-inflammatory (De Melo et al. 2005), antimelanogenic (Kim et al. 2018b) activities and inhibiting the formation of gallstones and kidney stones (Liu et al. 2017). Besides, schaftoside was also reported as a feeding inhibitor and resistance factor to brown planthopper (Stevenson et al. 1996). As an isomer of schaftoside, isoschaftoside (Isosch) was also found to be an allelochemical against the development of *Striga* (Hooper et al. 2010). Carlinoside (luteolin 6-*C*-glucosyl-8-*C*-arabinoside) from *Cajanus* plants shows antihepatitic and bilirubin solubilization activity (Das et al. 2018). Recently, both schaftoside and carlinoside were identified as active ingredients against COVID-19. In silico analysis regarded schaftoside as one of the top 10 among 318 phytochemicals that had significantly lower binding energy to Mpro (the main protease of SARSCoV-2) and ACE2 (angiotensin-converting enzyme 2) as compared to the reference molecule PRD_002214 (Joshi et al. 2020). Molecular docking indicated carlinoside was the top candidate against Mpro (Ettayapuram Ramaprasad et al. 2020; Joshi et al. 2020). Therefore, flavone *C*-arabinosides are expected to be a powerful weapon for potential treatment of SARSCoV-2.

Largely lagging behind the discovery and bioactivity assay of *C*-arabinosylated flavones, the in planta biosynthesis of *C*-arabinosides was occasionally studied (Putkaradze et al. 2021). At present, only a few *C*-glycosyltransferases (CGTs) that accommodating uridine-5′-diphosphate (UDP)-arabinose have been reported (Chen et al. 2018; Feng et al. 2021; He et al. 2019; Sun et al. 2020; Wang et al. 2020; Zhang et al. 2020). In our previous work, a group of gramineae CGTs was identified as glycosyltransferases utilizing UDP-glucose (UDP-Glc) and UDP-arabinose (UDP-Ara) for the *C*-glycosylation of phloretin and 2-hydroxynaringin (2-OHNar) (Sun et al. 2020). It is likely that the grass family plants have evolved two branches of CGTs, in which one group is more specialized for *C*-glucosylation (designated as clade A) and another is more relaxed to accept both UDP-Glc and UDP-Ara donors (designated as clade B). Correspondingly, the chemical diversity of flavone *C*-glycosides in Gramineae family does reflect the promiscuity of their CGTs, as both *C*-glucosyl and *C*-arabinosyl-carrying metabolites were frequently found in these grasses represented by rice (Besson et al. 1985; Melo et al. 2005; Talhi and Silva 2012). *Oryza sativa* (rice) is an important gramineae crop closely related to the life of billions of people. The leaves of *O. sativa* subsp. japonica accumulate a high proportion of flavone *C*-pentosylhexosides mainly represented by (iso)schaftoside and (iso)carlinoside (Sun et al. 2020). Such metabolite profiles indicate that CGTs from the rice may be excellent candidates for the production of flavone *C*-glycosides, especially flavone di-*C*-glycosides carrying hexosyl (i.e., glucosyl) and pentosyl (i.e., arabinosyl).

We previously discovered that the chromosome 6 of *O. sativa* subsp. indica (long-grain rice) harbors six tandem duplicated CGT-encoding genes, which is twice as many as those of *japonica* rice (Sun et al. 2020). Sequence analyses implied an expansion of clade B CGTs including 4 members (OsUGT708A1, OsUGT708A2, OsUGT708A39 and OsUGT708A40) (Additional File 1: Fig. S1). Genetic mechanism underlying the varietal differences of distinct rice genotype has been an attractive topic for long years, nevertheless there is still few studies mentioned the variance of rice *C*-glycoside spectrum and genes linked to such phenotypes. It is reasonable to hypothesize that the additional clade B CGTs in *indica* rice may play an important role in the formation of specific *C*-arabinosides, resulting in intraspecific difference.

At present, large-scale production of flavone *C*-glycosides, especially the rare flavone *C*-arabinosides is exclusively limited to plant extraction. Complex extraction processes and unsustainable source are great challenges to meet the ever-growing demand. Recently there have been some attempts on the production of flavone *C*-monoglucosides in heterologous chassis cells (Brazier-Hicks and Edwards 2013; Vanegas et al. 2018; Ito et al. 2014; Shrestha et al. 2018; Sun et al. 2020). However, as far as we know, there have been no reports of de novo biosynthesis of complex flavone (di)-*C*-glycosides with arabinosyl or other pentosyl moiety. With the development of synthetic biology, production of flavonoid glycosides by heterologous chassis cells become a promising alternative way to access these bioactive molecules at a much lower cost (Kim et al. 2015; Lim et al. 2015; Liu et al. 2018; Malla et al. 2013; Pandey et al. 2013; Pei et al. 2016; Schmidt et al. 2011; Shrestha et al. 2018; Simkhada et al. 2010). In this study, we proved the *C*-arabinosyl-transferring activity of rice CGTs on the mono-*C*-glucoside substrate nothofagin, followed by taking advantage of specific CGTs to realize the production of several di-*C*-glycosides including eight different *C*-arabinosides/xylosides. The strategy combining heterologous UDP-pentose supply, precursor supplement and fed-batch fermentation maximized the titer of rice-originated *C*-arabinosides to 20–110 mg/L in an *Escherichia coli* chassis for the time.

## Materials and methods

### Plant materials, chemicals

*Oryza sativa* subsp. japonica (cv. Nipponbare) and *Oryza sativa* subsp. indica (cv. Teqing) were grown in CAS Center for Excellence in Molecular Plant Science. Chemical standards including schaftoside (Sch), isoschaftoside (Isosch), phloretin (Phr), vitexin (Vit), isovitexin (Isovit), naringenin (Nar), *p*-coumaric acid (p-CA), vicenin-1 and vicenin-3 were purchased from Dalian Meilun Biotechnology Co., LTD (China). Nothofagin, apigenin 6,8-*C*-di-arabinoside (Api-di-*C*-Ara) and apigenin 6,8-*C*-di-xyloside (Api-di-*C*-Xyl) were prepared by our laboratory and confirmed by NMR analysis. UDP-glucose (UDP-Glc, Realtimes Biotechnology Co., Ltd., Beijing, China) and UDP-arabinose (UDP-Ara, CarboSource, U.S.A) were used as sugar donors in the enzymatic assays.

### Metabolic analysis of leaves of two rice cultivars

The frozen leaves of two rice cultivars were extracted as previously described (Sun et al. 2020). Briefly, the crude examples extracted by 75% methanol were concentrated, re-suspended in water and extracted three times with n-butanol. The organic layer was combined and evaporated to dryness. All samples were analyzed by high-performance liquid chromatography (HPLC) on an Ultimate 3000 HPLC system (ThermoFisher Scientific) with an Ecosil 120-5-C18 column (φ4.6 × 250 mm, 5 μm). The compounds were separated by water (containing 0.1% formic acid, solvent A) and acetonitrile containing 0.1% formic acid, solvent B) at a flow rate of 1.0 mL/min. The samples were eluted under a linear gradient condition: 0.0–16.0 min, 10%–25% B; 16.0 min–30.0 min, 25%–100% B; 30.0 min–35.0 min, 100% B. Ultra-performance liquid chromatography (UPLC)–high-resolution (HR)-mass (MS)/MS were acquired using Q Exactive hybrid quadrupole-Orbitrap mass spectrometer (Thermo Scientific, U.S.A.) equipped with an Acquity UPLC BEH C18 column (φ2.1 × 50 mm, 1.7 μM, Waters, U.S.A.). A linear gradient was set as follows: 0.0–10.0 min, 5%–95% acetonitrile (0.1% formic acid) in H_2_O; 10.0–12.5 min, keep 95% acetonitrile; 12.5–15.0, re-equilibrate to the initial condition. The flow rate was 0.25 mL/min. The mass acquisition was performed in negative ionization mode with full scan (50–1000).

### Expression of *C*-glycosyltransferases

The genomic DNA (gDNA) of *O. sativa* indica was extracted by the Plant Genomic DNA Kit (Tiangen, Beijing). *OsUGT708A1* and *OsUGT708A40* were amplified directly from the gDNA by PCR using PrimeSTAR Max DNA polymerase (Takara, Japan). *OsUGT708A2* and *OsUGT708A39* were synthesized and codon-optimized by Genscript Co. Ltd. (Nanjing, China). The rice *CGTs* were inserted into *Nde*I/*Not*I-double digested pET28a via plus One step PCR Cloning Kit (NovoRec, Shanghai, China) (Additional file 1: Table S1) and transformed into *E. coli* BL21(DE3) for recombinant expression. Positive clones were grown overnight in 2 mL Luria–Bertani (LB) media and inoculated into 100 mL of fresh LB medium. When the OD_600_ reached 0.5–0.7, 0.1 mM isopropyl β-d-1-thiogalactopyranoside (IPTG) was used to induce the protein expression at 16 °C for 20 h. The cells were collected by centrifugation (6000 rpm, 5 min) and lysed by using a sonication homogenizer (50 W, five cycles). The crude protein extracts were stored at − 20 °C for subsequent purification. Ni NTA Magarose Beads (Shanghai Chuzhi Biological Technology, Shanghai, China) was used to purify the His_6_-tagged protein.

### In vitro enzymatic assay of CGTs

A typical enzymatic assay was performed in a 100 μL aliquot of reaction mixture containing buffer A (100 mM NaCl, 20 mM Tris–HCl, pH 8.0), 200 μM UDP-arabinose, 100 μM nothofagin and 25 μg purified enzymes. The reaction mixtures were incubated at 37 °C for 2 h. One hundred microliter of methanol was added to quench the reaction. The mixtures were centrifuged (12,000 rpm) for 15 min and subjected to HPLC analyses. Separation was achieved on a C18 column [SilGreen ODS column (φ4.6 × 250 mm, S-5 μM), Greenherbs Co., Ltd., Beijing, China] with a flow rate of 1 mL/min at 40 °C. Mobile phases contained acetonitrile (0.1% formic acid, solvent A) and H_2_O (0.1% formic acid, solvent B) under a linear gradient elution: 0–20 min, 5% to 100% A in B, 100% A maintained for 5 min. The absorption was monitored at λ = 280 nm and 340 nm.

### Reconstruction of *C*-arabinoside pathway

*OsUGT708A1* was inserted into pCZ86 (pET28a harboring *PhUGT708A43*, Additional file 1: Table S1) between the *Not*I site via ClonExpress II One Step Cloning Kit (Vazyme, Nanjing, China), resulting in pCZ191. In order to identify an optimal combination for UDP-arabinose biosynthesis, we cloned *SmUxs1*, *SmUxs2* and *SmUxe* from *Sinorhizobium meliloti* 1021. AxyPrep Bacterial Genomic DNA Miniprep Kit (Axygen, USA) was used to extract the gDNA of *S. meliloti*. *SmUxs1*, *SmUxs2* and *SmUxe* were amplified by PCR using PrimeSTAR Max DNA polymerase (Takara, Japan) with gene-specific primers (Additional file 1: Table S2). We first inserted *SmUxs1* and *SmUxs2* into *BamH*I-digested pCZ191 to give pCZ192-1 and pCZ192-2, respectively. *SmUxe* was further introduced into the *BamH*I site of pCZ192-1 and pCZ192-2 via ClonExpress II One Step Cloning Kit to accomplish pCZ193-1 and pCZ193-2. The cassette of *SmUxs1-SmUxe* was amplified using pCZ192-1 as template and inserted into the *Not*I site of pCZ165 (pET28a harboring *OsUGT708A40*, Additional file 1: Table S1) to accomplish pCZ194 which was ready for apigenin *C*-arabinoside production. The whole sequence of pCZ194 excepted *SmUxe* was amplified by reverse PCR to give pCZ195. For the de novo production of flavone *C*-arabinoside and flavone *C*-xyloside from tyrosine, the assembled glycosylation modules (pCZ193-1/pCZ193-2/ pCZ192-1/pCZ194/pCZ195) or empty vector pET28a were co-transferred with pYH055 (Li et al. 2019) and pCZ201 (Sun et al. 2020) into *E. coli* BL21(DE3) to give strain sCZ113, sCZ114, sCZ115, sCZ118, sCZ119 and sCZ110 (as a negative control).

### Fermentation of *C*-arabinosylated flavones

For flask-shake fermentation, the seeds were precultured at 37 °C in Luria broth (LB) medium overnight and then inoculated (1:100) into MOPS minimal medium supplemented with 5 g/L glucose. After the OD_600_ reached to 1.0, IPTG (0.1 mM) and tyrosine (0.5 g/L) was added to the cultures. Subsequently, the cultures were incubated at 22 °C, 250 rpms and maintain for 96 h.

For bioreactor fermentation, the seeds were precultured at 37 °C in LB medium overnight and then inoculated (1:100) into 100 mL LB medium for 5 h. Then all 100 mL seeds were inoculated into a 5-L bioreactor (Biostat B plus, BioSartorius Stedim Biotech, Germany) at 37 °C, which contained 1 L M9 minimal media (15.7 g/L K_2_HPO_4_·3H_2_O, 4.2 g/L KH_2_PO_4_, 2 g/L (NH_4_)_2_SO_4_, 1.8 g/L citric acid, 1.2 g/L MgSO_4_·7H_2_O, 0.5 g/L yeast extract) with 20 g/L glucose, 5 mL/L of trace metal solution and 100 μL antifoam 204. The trace metal solution contained 0.5 M HCl, 10 g FeSO_4_·7H_2_O, 2 g CaCl_2_, 2.2 g ZnSO_4_·7H_2_O, 0.5 g MnSO_4_·4H_2_O, 1 g CuSO_4_·5H_2_O, 0.1 g (NH_4_)_6_Mo_7_O_24_·4H_2_O, and 0.02 g Na_2_B_4_O_7_·10H_2_O per liter of solution. When the OD_600_ increased to 10, IPTG (0.5 mM) and tyrosine (1 g/L) was added to the cultures and the pH, temperature and dissolved oxygen (DO) were automatically maintained at 7.0, 22 °C and 30% (v/v). The feeding solution contained 500 g/L glucose, 10.7 g/L (NH_4_)_2_SO_4_, 12 g/L MgSO_4_·7H_2_O, 5 g/L yeast extract and 5 mL/L trace metal solution.

All the culture samples (500 μL) were extracted by 500 μL n-butanol for three times. The combined supernatant was evaporated under vacuum and dissolved in 100 μL methanol; 20 μL was injected for HPLC and UPLC–MS/MS analysis. Condition of LC–MS/MS was identical to that described above.

### Identification and isolation of *C*-glucosylated flavones

In order to confirm the production of representative *C*-glycosylated flavones, we isolated three products (Api-di-*C*-Ara, Api-di-*C*-Xyl, Chr-di-*C*-Ara) from 1 L fermentation broth of strain sCZ118. The fermentation broth was all gathered by centrifuged 6000 rpm, the liquid supernatant was subjected to Diaion HP20 (Mitsubishi Co., Ltd., 1L) and eluted with increasing gradient of ethanol (from 20 to 100%) in H_2_O. Two fractions eluted with 60% and 80% ethanol, which contain the Api-di-*C-*Ara and minor glycosides were combined and evaporated. The residues were dissolved in 20% methanol, subjected to MCI-gel (Mitsubishi Co., Ltd., 250 mL) and eluted with increasing gradient of methanol (from 20 to 100%) in H_2_O. A fraction eluted with 60% methanol, which mainly contains Api-di-*C*-Ara was evaporated and the residue was dissolved in 10% methanol. The dissolved component was subjected to ODS silica gel column (YMC-gel ODS-A-HG, 12 nm, S-50 μm, 100 mL, YMC CO., Ltd., Japan) and eluted with increasing gradient of methanol (from 10 to 100%) in H_2_O. Two fractions eluted with 30% and 40% methanol were was further purified repeatedly by ODS silica gel column to yield purified Api-di-*C*-Ara, Api-di-*C*-Xyl and Chr-di-*C*-Ara. ^1^H, ^13^C and 2D NMR spectra (Additional File 1: Figs. S6, S7, S8) were recorded at 80 °C on AVANCE-500 (500 MHz for ^1^H) spectrometer (Bruker BioSpin, Rheinstetten, Germany). The chemical shifts (ppm) were referenced to the solvent (DMSO-*d*_6_) peaks at δ_H_ = 2.50 ppm and δ_C_ = 39.5 ppm.

## Results and discussion

### Rice CGTs responsible for varietal di-*C*-glycosides

**Fig. 1:**
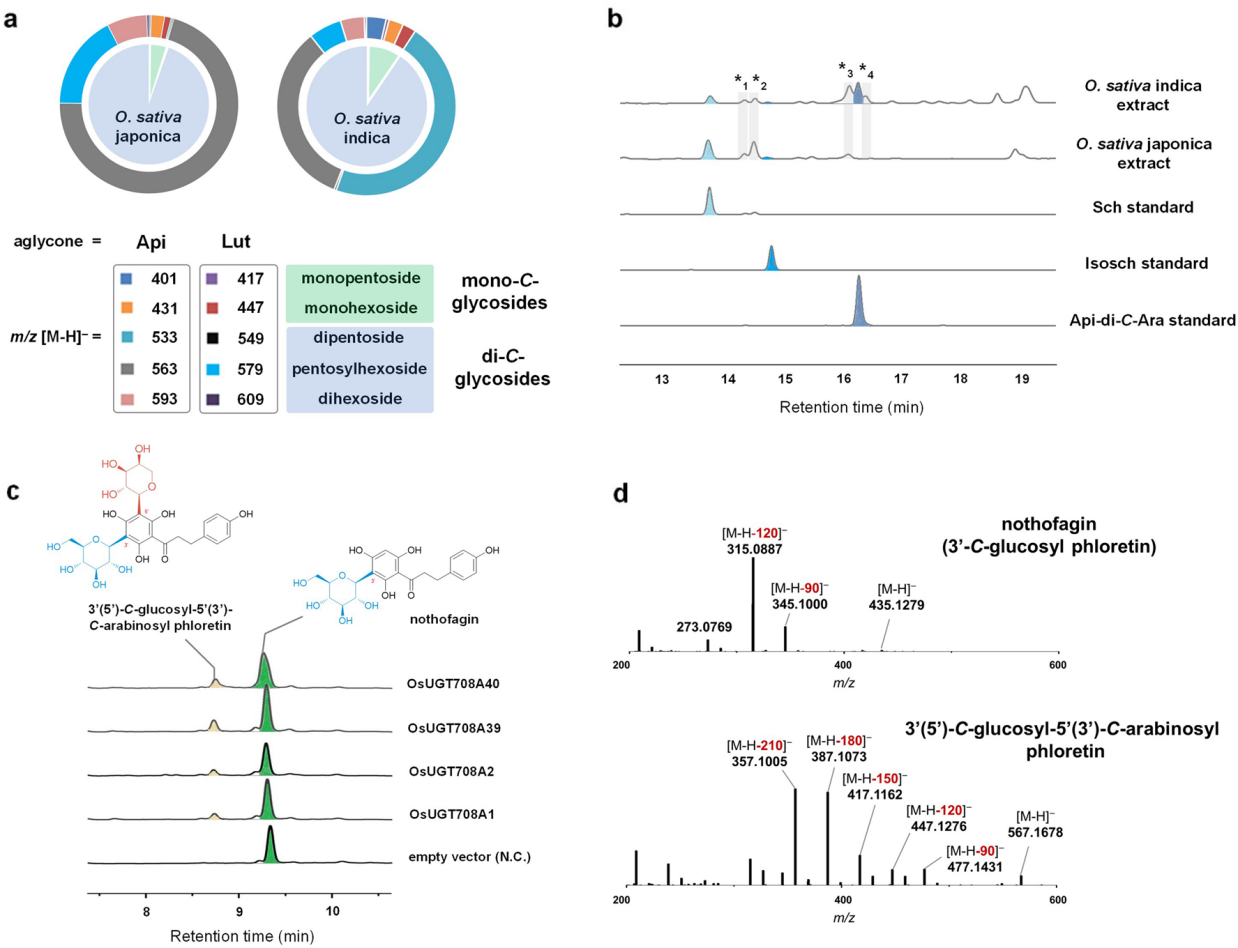
Characterization of rice CGTs responsible for di-*C*-glycosides biosynthesis. **a** Composition of *C*-glycosides in different rice. The pie chart indicated the percentage of mono-*C*-glycosides and di-*C*-glycosides. The doughnut chart indicated individual *C*-glycosides of apigenin (Api) and luteolin (Lut). The ion abundance corresponding to [M–H]^–^ peaks was calculated. **b** HPLC analysis of rice metabolites recorded on 280 nm. For minor diglycosides compound *1–*4, see Additional file 1: Fig S2. **c** HPLC chromatograms of the reactions of rice Clade B CGTs with UDP-Ara and nothofagin. **d** MS/MS fragmentation of nothofagin and its *C*-arabinosylated product

In our ongoing investigation of Gramineae CGTs, we first compared the differences of *C*-glycoside spectrum between two rice subspecies (*japonica* vs *indica*) in detail (Fig. 1a). The rice leaves were extracted and subjected to LC–MS/MS analysis. Because most of the flavone *C*-glycosides in rice share the common aglycone apigenin (Api) or luteolin (Lut) (Besson et al. 1985), we determined to focus on five representative groups of Api/Lut-*C*-glycosides, corresponding to monopentosides, monohexosides, dipentosides, pentosylhexosides and dihexosides. Both rice varieties were found to predominantly produce di-*C*-glycosides (96% in *O. sativa* japonica, 91% in *O. sativa* indica, Fig. 1a), however, the composition of diglycosides differed drastically. The *japonica* rice particularly accumulated apigenin *C*-pentosylhexoside (corresponding to *m/z* [M–H]^–^ = 563.1), whereas the *indica* rice majorly produced apigenin di-*C*-pentoside (corresponding to *m/z* [M–H]^–^ = 533.1) besides apigenin *C*-pentosylhexoside (Fig. 1a). The most abundant diglycosides were verified as schaftoside (Sch) and apigenin 6,8-di-*C*-arabinoside (Api-di-*C*-Ara) as referenced to the authentic samples (Fig. 1b). In accordance with the previously recorded metabolic profiling (Kim et al. 2018a; Ramarathnam et al. 1989; Yang et al. 2016), glucosyl and arabinosyl residues seem to be the representative hexose and pentose present in rice. Other minor flavone diglycosides were proposed to be *C*-pentosylhexosides, di-*C*-pentosides and di-*O*-glycosides with diverse sugar-linkages (Additional File 1: Fig. S2). It is also worthy to note that *C*-glycosides of apigenin are generally more abundant than those of luteolin, regardless of the glycosylation patterns (Fig. 1a).

**Fig. 2:**
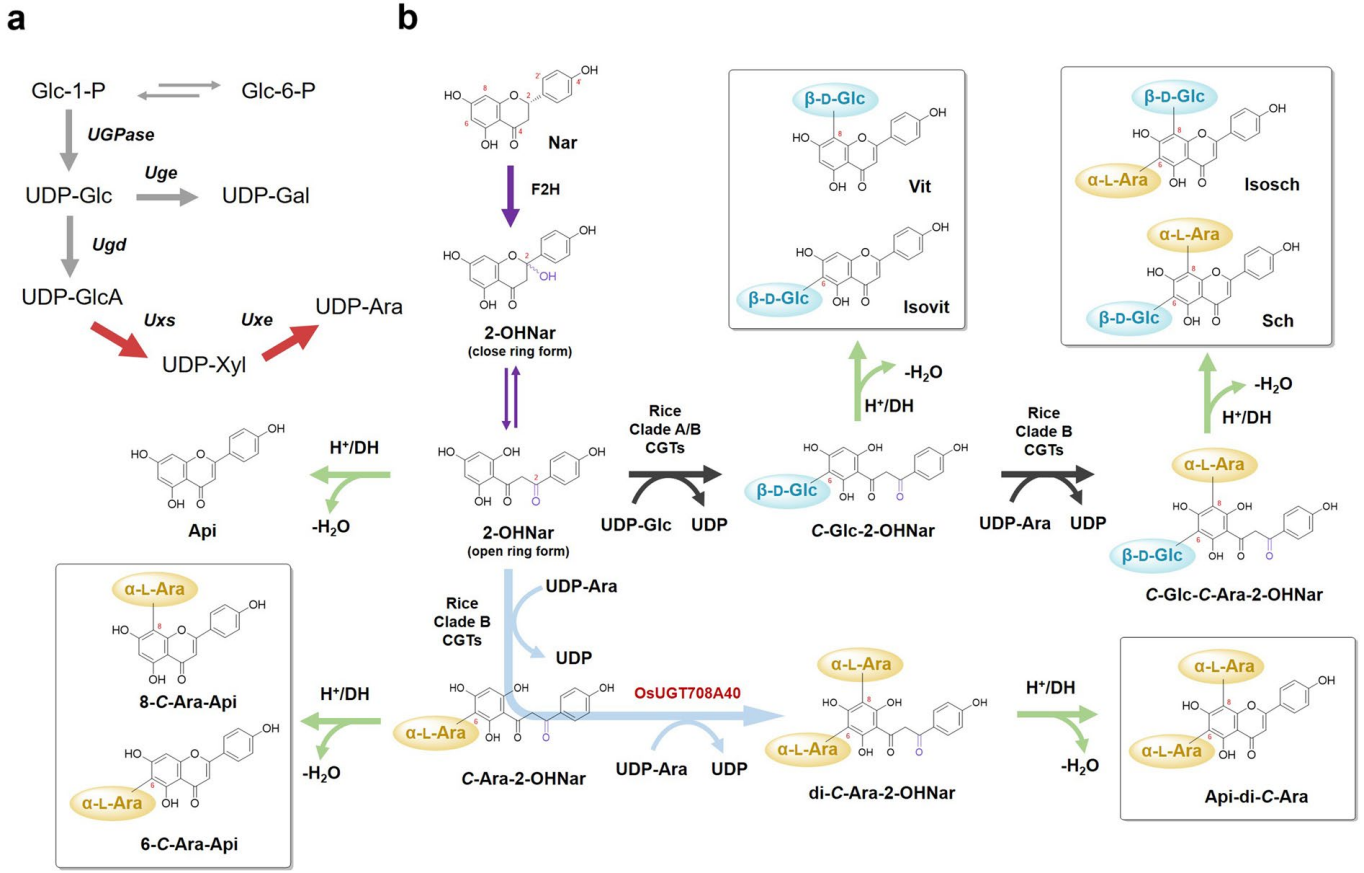
A proposed biosynthetic network of *C*-glycosylated apigenin in rice. **a** UDP-arabinose (Ara) is generated from glucose-1-phosphate (Glc-1-P) via UDP-glucose (Glc) and UDP-xylose (Xyl). Grey arrows represent the native metabolism in *E. coli*, while the bold red arrows represent an introduced heterologous UDP-Ara pathway. Glc-6-P glucose-6-phosphate, Gal galactose, GlcA glucuronate, UGPase UDP-glucose pyrophosphorylase, Uge UDP-glucose 4-epimerase, Ugd UDP-glucose 6-dehydrogenase, Uxs UDP-xylose synthase/UDP-glucuronic acid decarboxylase, Uxe UDP-xylose 4-epimerase. **b** A proposed *C*-glycosylated apigenin pathway starting from naringenin (Nar). Purple arrows represent 2-hydroxylation by flavanone 2-hydroxylases (F2H) and equilibrium of ring-open/closed 2-hydroxynaringenin (2-OHNar). Black arrows indicate two-step glycosylation reactions in (iso)schaftoside biosynthesis (Sch). Glycosylation reactions forming apigenin 6,8-di-*C*-arabinoside (Api-di-*C*-Ara) are indicated in sky blue arrows. After the formation of *C*-glycosylated intermediates, dehydration reactions (green arrows) occur spontaneously in acidic solvent or by dehydratases (DH), producing a mixture of 6-*C*- or 8-*C*-isomers. Api apigenin, 2OHNar 2-hydroxylnaringenin, Vit vitexin, Isovit isovitexin

In our previous work, both Clade A and Clade B CGTs from grass family were proved to be able to recognize the non-sugar-bearing aglycones [i.e., phloretin, 2-hydroxynaringenin (2-OHNar) (Sun et al. 2020), resulting in majorly mono-*C*-glucosides and arabinosides. It remains unclear that in rice how the aglycones undergo two steps of *C*-glycosylation to reach di-*C*-glycosides bearing different sugars (for example, schaftoside and isoschaftoside). According to the existing knowledge of flavone *C*-glycoside biosynthesis (Putkaradze et al. 2021), we proposed a biosynthetic pathway in which the rice CGTs collaborate to first install a *C*-glucosyl (mainly by Clade A CGTs) on the precursor (2-OHNar), followed by addition of a second *C*-arabinosyl group (mainly by Clade B CGTs) (Fig. 2, black arrows). To verify whether Clade B CGTs could accept monoglucoside substrates like *C*-glucosyl-2-hydroxynaringenin (*C*-Glc-2-OHNar), we expressed the His_6_-tagged OsUGT708A1, OsUGT708A2, OsUGT708A39, and OsUGT708A40 in *E. coli* BL21(DE3) and tested their activities toward nothofagin (3′-*C*-glucosyl phloretin, a relatively stable analogue of *C*-Glc-2-OHNar) in in vitro enzymatic assays. In the presence of UDP-Ara, nothofagin was converted to a new product with *m/z* = 567.2 (Fig. 1c, d). The characteristic fragment ions such as [M–H–150]^–^/[M–H–180]^–^/[M–H–210]^–^ in MS/MS spectrum clearly revealed a hybrid pattern of *C*-pentosylation and *C*-hexosylation in good agreement with the structure of 3′-*C*-glucosyl-5′-*C*-arabinosyl phloretin. These results suggested that rice Clade B CGTs could catalyze the arabinosyl-transferring reaction of *C*-monoglucoside substrate, which was a key step in the biosynthesis of *C*-pentosylhexosides like schaftoside or isoschaftoside. Recently, the dissection of schaftoside pathway in other plants (mainly represented by dicot plants like *Scutellaria baicalensis* and *Nelumbo nucifera*) also supported the above results (Feng et al. 2021; Wang et al. 2020), which shows the generality of di-*C*-glycoside pathway in higher plants.

Unlike *O. sativa* japonica that accumulates *C*-pentosylhexoside, *O. sativa* indica produces a large amount of apigenin di-*C*-pentoside occupying 46% of the total flavone *C*-glycosides (Fig. 1a). There is also an increase of apigenin mono-*C*-pentoside (corresponding to *m/z* [M–H]^–^ = 401.1). This is probably due to the three additional clade B CGTs (OsUGT708A1, OsUGT708A39 and OsUGT708A40) only present in *indica* rice (Fig. 1), which can utilize UDP-Ara to convert 2-OHNar to *C*-Ara-2-OHNar (alternatively, phloretin to *C*-arabinosyl phloretin) (Sun et al. 2020). In particular, among the Clade B CGTs, OsUGT708A40 is a unique di-*C*-arabinosyltransferase that catalyzes a tandem *C*-arabinosylation reaction (Sun et al. 2020). We proposed that OsUGT708A40 was a key di-*C*-arbinosyltransferase responsible for the formation of apigenin 6,8-di*-C*-arabinoside (Fig. 2, light blue arrows).

### Introduction of UDP-arabinose and UDP-xylose supply allowed de novo biosynthesis of schaftoside, isoschaftoside, vicenin-1 and vicenin-3

**Fig. 3:**
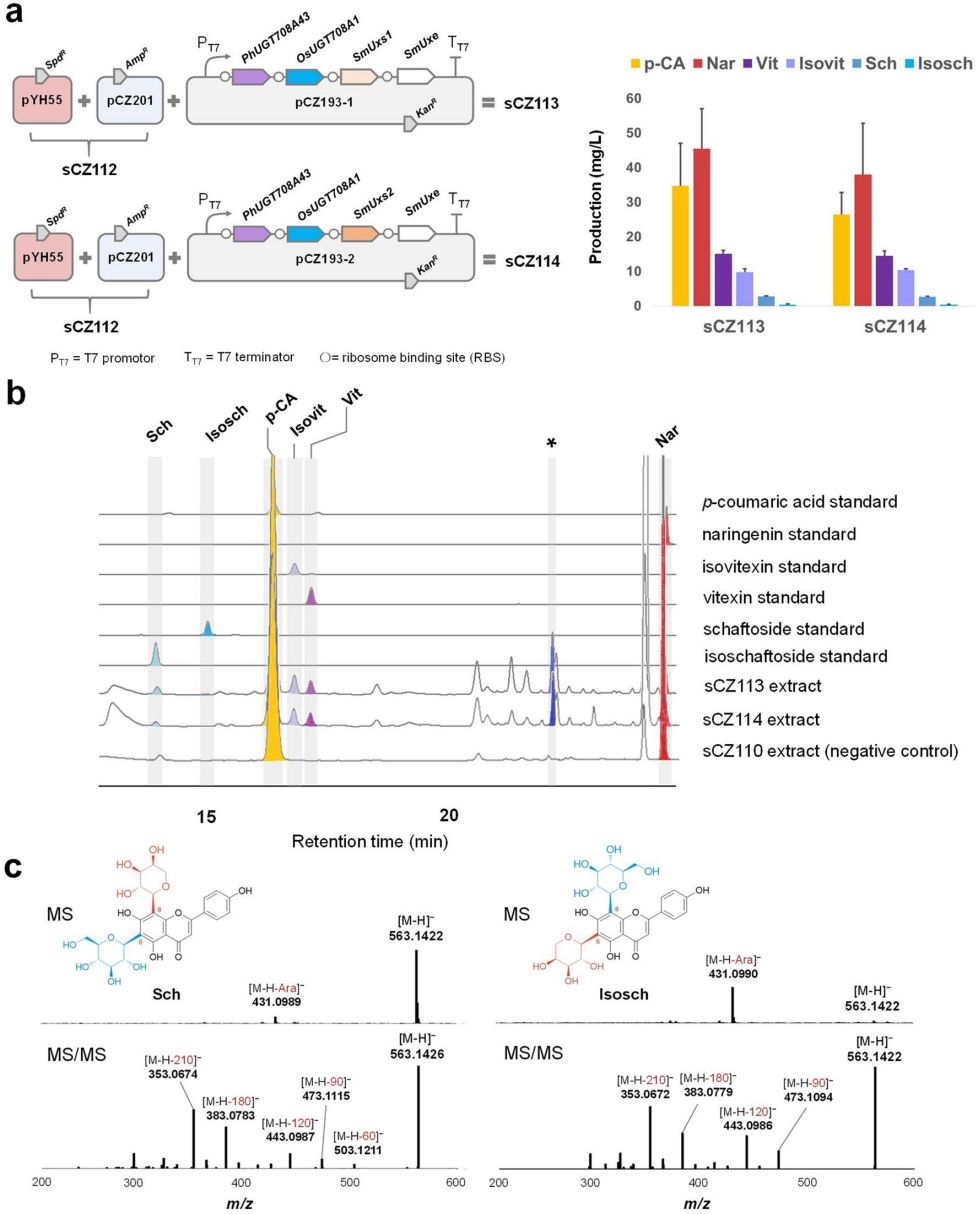
De novo biosynthesis of schaftoside. **a** Reconstitution of schaftoside pathway in *E. coli* chases. pYH55 (Li et al. 2019) is assembled for naringenin (Nar) production and pCZ201 (Sun et al. 2020) harbors cytochrome P450 module for 2-hydroxylnaringenin (2-OHNar) production. Fermentation of sCZ113 and sCZ114 revealed similar productivity. **b** HPLC chromatography of the extract of sCZ113. Standard samples were also analyzed for comparison. The peak indicated in asterisk was temporarily identified as apigenin 6(8)-*C*-arabinoside. UV absorbance at 280 nm was monitored. (**C**) MS and MS/MS spectra of schaftoside (Sch) and isoschaftoside (Isosch) present in the extract of sCZ113

To further prove our proposed pathway (Fig. 2a, b) and achieve de novo biosynthesis of bioactive di-*C*-glycosides, we selected the fast growing and genetically amenable *E. coli* as a suitable chassis for pathway reconstitution. The previously constructed sCZ112 harboring pYH55 (Li et al. 2019) and pCZ201 (Sun et al. 2020) for optimized 2-hydroxynaringenin production was used as the starting strain. In order to realize the heterologous biosynthesis of *C*-pentosylhexoside like schaftoside, we first assembled a di-*CGT* cassette containing *PhUGT708A43* (an excellent coding *C*-monoglucosylating enzyme from moso bamboo (Sun et al. 2020) for the first step of glucosylation) and *OsUGT708A1* (for the subsequent *C*-arabinosylation) under T7 promoter (Fig. 3a).

A major difficulty for the biosynthesis of arabinosides in *E. coli* is the absence of native UDP-arabinose supply. To solve this problem, we introduced SmUxs (UDP-xylose synthase) and SmUxe (UDP-xylose 4-epimerase) from *Sinorhizobium meliloti* 1021 (Gu et al. 2011) to enable the metabolism from UDP-glucose to UDP-arabinose (Fig. 2a). Two *SmUxs* homologues (*SmUxs1* and *SmUxs2*), sharing only 57.3% amino acid identity, were, respectively, ligated downstream to the *PhUGT708A43*-*OsUGT708A1* cassette and further assembled with *SmUxe* to give pCZ193-1 and pCZ193-2 ready for the production of schaftoside (Fig. 3a). After transferring pCZ193-1 or pCZ193-2 into sCZ112 (resulting in strain sCZ113 and sCZ114, respectively), we successfully detected 2.75 mg/L schaftoside (Sch) and 0.43 mg/L isoschaftoside (Isosch) in sCZ113 broth through 72-h fermentation in MOPS media (Fig. 3b). The pathway intermediates like vitexin (Vit, 15.14 mg/L), isovitexin (Isovit, 9.78 mg/L), naringenin (Nar, 45.54 mg/L) and *p*-coumaric acid (p-CA, 34.79 mg/L) were also observed (Fig. 3a, b). All the products were identified through comparison with authentic samples in HPLC analysis (Fig. 3b) and high-resolution (HR) MS/MS spectroscopic data (Fig. 3c, Additional File 1: Fig. S3). On the other hand, 2.67 mg/L Sch and 0.41 mg/L Isosch were detected in sCZ114. The accumulation of Vit, Isovit and Nar reached 14.52 mg/L, 10.42 mg/L and 38.01 mg/L. A similar productivity of Sch/Isosch and no significant difference of accumulation pattern of intermediates between SmUxs1 and SmUxs2 (Fig. 3a), therefore we used SmUxs1 for further experiments.

Since UDP-xylose is an upstream precursor of UDP-arabinose (Fig. 2a), we proposed that flavone *C*-xylosides might be generated in a truncated pathway containing biosynthetic genes fitting just for UDP-xylose biosynthesis (Additional File 1: Fig. S4). Therefore, we also try to achieve the production of vicenin-1 (apigenin 6-*C*-xylosyl-8-*C*-glucoside, Vic-1) and vicenin-3 (apigenin 6-*C*-glucosyl-8-*C*-xyloside, Vic-3). After transferring pCZ192-1 (harbors the cassette of *PhUGT708A43*-*OsUGT708A1-SmUxs1*) into sCZ112 (resulting in strain sCZ115), we detected a trace amount of Vic-1 (0.09 mg/L) and Vic-3 (0.28 mg/L) in 72 h fermentation (Additional File 1: Fig. S5), which is a much lower titer compared to that of Sch and Isosch. This result indicated that UDP-xylose might not be a favorite sugar donor of OsUGT708A1. To the best of our knowledge, di-*C*-glycosides like Sch, Isosch, Vic-1 and Vic-3 were synthesized in heterologous chassis cells for the first time. These results indicated the feasibility of de novo production of *C*-arabinoside and *C*-xyloside in *E. coli*.

### De novo biosynthesis of apigenin di-*C*-arabinoside and minor *C*-pentosides

Flavone compounds bearing multiple *C*-pentosyl (for example, arabinosyl, xylosyl) residues are uncommon natural products. To further expand the diversity of flavone *C*-glycosides, we attempted to construct an artificial pathway in *E. coli* for the production of apigenin di-*C*-arabinoside and other minor *C*-pentosides. The biosynthesis of specific di-*C*-arabinosides requires efficient di-*C*-glycosyltransferase preferring UDP-Ara, as well as a heterologous UDP-Ara-synthesizing module above-mentioned. OsUGT708A40 was selected as a proper enzymatic part since it was identified as the only di-*C*-arabinosyltransferase in rice (Sun et al. 2020). Due to the close similarity of UDP-Ara and UDP-xylose (UDP-Xyl), we predicted that OsUGT708A40 might also promiscuously consume UDP-Xyl for some minor *C*-xyloside production.

**Fig. 4:**
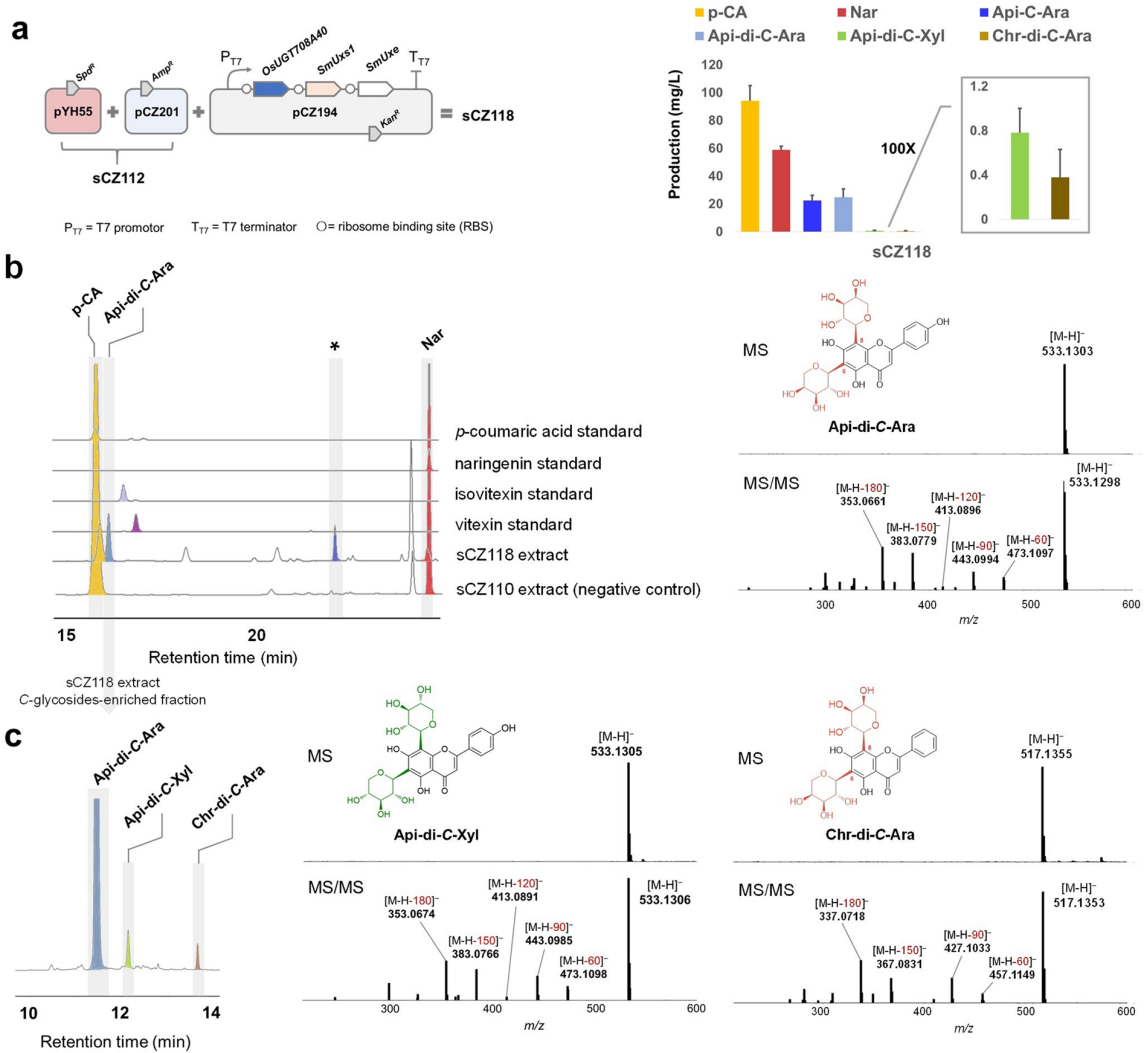
De novo biosynthesis of uncommon *C*-glycosides. **a** pYH55 (Nar module) (Li et al. 2019), pCZ201 (cytochrome P450 module) (Sun et al. 2020) and pCZ194 (arabinosylation module) were co-expressed to reconstitute apigenin di-*C*-arabinoside (Api-di-*C*-Ara) pathway in *E. coli* chases. **b** HPLC analysis of the extract of sCZ118 and HR-MS fragmentation of Api-di-*C*-Ara. The peak indicated in asterisk was temporarily identified as apigenin 6(8)-*C*-arabinoside. UV absorbance at 280 nm was monitored. **c** Characterization of minor *C*-glycosides co-eluted with Api-di-*C*-Ara. HR-MS and MS/MS indicated the presence of apigenin di-*C*-xyloside (Api-di-*C*-Xyl) and chrysin 6,8-*C*-di-arabinoside (Chr-di-*C*-Ara)

The construct pCZ194 harboring *OsUGT708A40-SmUxs1-SmUxe* cassette was transformed into sCZ112 to give strain sCZ118 (Fig. 4a). After 72 h fermentation, we detected the emergence of new products characteristic of *C*-pentosides instead of *C*-glucosides (i.e., Vit/Isovit) in the fermentation media. We quantified these products using vitexin and vicenin-2 as internal standards. The major peak with a retention time (R_t_) = 16.02 min was identified as apigenin 6,8-*C*-di-arabinoside (Api-di-*C*-Ara) based on both LC–MS/MS and NMR evidence (Fig. 4b, Additional File 1: Fig. S6). The ^1^H NMR spectrum of Api-di-*C*-Ara recorded at 80 ℃ clearly revealed the doublets of aglycone H2’, 6’ and two anomeric protons of sugars (Additional File 1: Fig. S7). The α-l-arabinosyl was clarified though correlations between Ara-H1, Ara-H5a and Ara-H4 observed in 2D NOESY spectrum (Additional File 1Fig. S6f). In addition to Api-di-*C*-Ara, we also detected two minor *C*-pentosides which are supposed to be apigenin 6,8-*C*-di-xyloside (Api-di-*C*-Xyl) and chrysin 6,8-*C*-di-arabinoside (Chr-di-*C*-Ara), according to the HR-MS/MS and NMR analyses (Fig. 4c, Additional File 1: Fig. S8). Unfortunately, we didn’t succeed in obtaining and distinguishing apigenin 6-*C*-arabinoside or 8-*C*-arabinoside [Api-(6/8)-*C*-Ara], since they decomposed quickly in the solvent (Additional File 1: Fig. S9). Without extra optimization, the titer of Api-di-*C*-Ara, Api-di-*C*-Xyl, Chr-di-*C*-Ara and nascent Api-*C*-Ara reached 24.89 mg/L, 0.78 mg/L, 0.38 mg/L, 21.15 mg/L, respectively.

Similarly, we also constructed pCZ195 specific for Api-di-*C*-Xyl production (Additional File 1: Fig. S8). As expected, after 72 h fermentation, we detected 3.26 mg/L Api-di-*C*-Xyl as major product with no flavone *C*-arabinosides accumulated (Additional File 1: Fig. S10). While compared with the productivity of Api-di-*C*-Ara (24.89 mg/L) in sCZ118, the production of Api-di-*C*-Xyl was much lower. This could also be explained by the substrate preference of OsUGT708A40 to UDP-Ara rather than to UDP-Xyl.

### Fed-batch fermentation of *C*-arabinosides

**Fig. 5:**
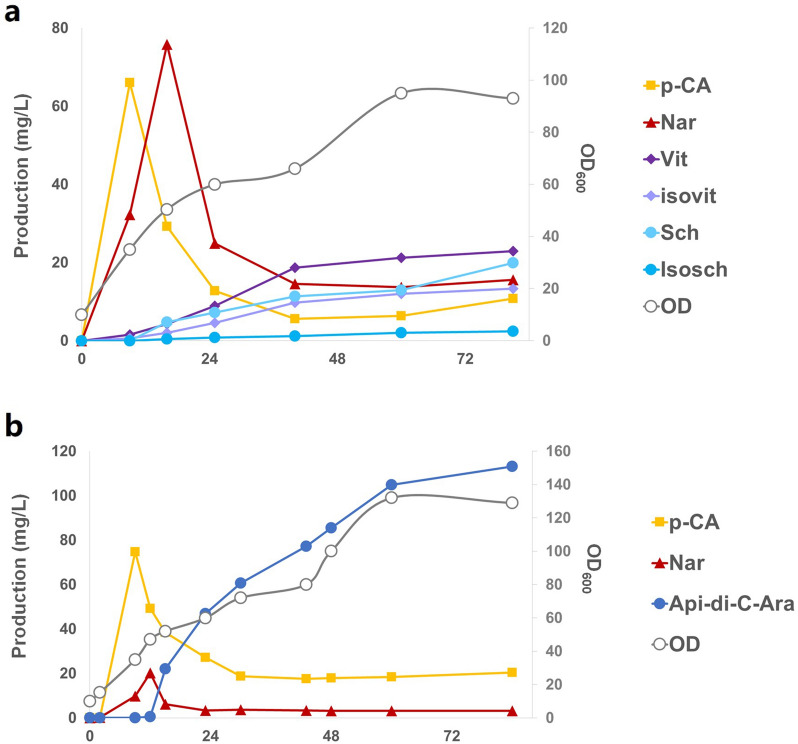
Fed-batch fermentation of *C*-arabinosides. **a** Fed-batch fermentation of sCZ113 in 5-L bioreactor. **b** Fed-batch fermentation of sCZ118 in 5-L bioreactor

To achieve a large-scale production and verify the scalability of our *C*-glycoside-producing strains, we performed scale-up fermentation of sCZ113 and sCZ118 in a 5-L bioreactor. The minimal M9 media with 20 g/L glucose was used as basal culture medium and 500 g/L glucose was used as supplementary medium. During the fermentation process of sCZ113, *p*-coumaric acid (p-CA) rapidly accumulated to 66.1 mg/L at 9 h (after induction) at the first stage and then rapidly decreased (Fig. 5a). Afterwards, naringenin (Nar) accumulated to 75.8 mg/L at 16 h until it was consumed. Vitexin (Vit) and isovitexin (Isovit) appeared at about 9 h, and schaftoside (Sch)/isoschaftoside (Isosch) appeared later (at approximate 16 h). After 81 h fermentation, production of Sch and Isosch reached 19.87 mg/L (7.2-fold compared to flask-shake) and 2.41 mg/L (5.6-fold compared to flask-shake) with 22.87 mg/L Vit and 13.32 mg/L Isovit left.

During the fermentation process of sCZ118, p-CA (74.8 mg/L) and Nar (20.29 mg/L) first rapidly accumulated to the maximum within 9 h (Fig. 5b). After 84 h fermentation, production of Api-di-*C*-Ara reached to 113.16 mg/L (4.7-fold compared to flask-shake). The results confirm that our fermentation process could be scaled up controllably and productively, which proved that fed-batch fermentation was beneficial to the accumulation of downstream glycosylated products. Our engineered *E. coli* system has the ability to supply enough UDP-Ara for large production of flavone *C*-arabinosides, which displays great industrial potential.

## Conclusion

Rice (*Oryza sativa*) is one of the most important crops feeding more than 3 billion of people. The subspecies *indica* and *japonica* are two main varieties of the cultivated rice. Investigation of the difference between two close subspecies has always been an interesting topic. In this research, we discovered dramatic difference of the *C*-glycosylated flavones, especially the metabolites containing arabinosyls occurring in two rice subspecies. Schaftoside featuring a hybrid *C*-glucosylation/*C*-arabinosylation is the most abundant diglycoside metabolite in *japonica* rice. In our previous work, *japonica* rice-originated OsUGT708A2, OsUGT708A3 and OsUGT708A4 were all identified as *C*-glucosyltransferases acting on aglycone substrates (phloretin, 2OH-Nar). Through the analyses of enzymatic function, we demonstrated this time that OsUGT708A2 (belongs to Clade B) was also able to *C*-arabinosylate monoglucoside substrates, which might explain the formation of flavone *C*-pentosylhexosides like schaftoside and isoschaftoside. This result is in good agreement with the recent work reported by (Wang et al. 2020). Due to the absence of other mono- and di-*C*-arabinosyltransferases in *japonica* rice, mono- and di-*C*-arabinoside was barely detected. In comparison, *O. sativa* indica produces apigenin di-*C*-arabinoside as the major flavone *C*-glycoside. We proposed that the specific CGTs in *indica* rice (OsUGT708A1, OsUGT708A39 and OsUGT708A40) influenced the accumulation pattern of flavone *C*-glycosides and caused diverse metabolisms in different rice cultivars*.* In particular, OsUGT70A40 may catalyze tandem *C*-arabinosylation to form di-*C*-arabinoside. Such different metabolic profiling was also observed in minor products of rice, as *japonica* rice accumulated more chrysoeriol *C*-glucosyl-*C*-arabinoside (compound *2) than *indica* rice did, while chrysoeriol di-*C*-arabinoside (compound *4) was only found in *indica* rice (Fig. 1b and Additional File 1: Fig. S2). Overall, hybrid *C*-glucosylation/*C*-arabinosylation is more common in *japonica* rice and di-*C*-arabinosylation is the major flavone decoration in *indica* rice. The expansion of rice clade B CGTs represents a good example of how plants evolve new enzymes to diversify their particular chemicals, suggesting the importance of *C*-glycosyltransferases in plant metabolism.

In nature, the grass family plants produce a highly complex mixture of *C*-glycosides consisting of *C*-pentosylhexoside, mono-*C*- and di-*C*-pentosides. It is time-consuming to isolate and purify these compounds, which perhaps hinders the evaluation of their potential pharmaceutical and nutraceutical values. Due to the rarity of *C*-arabinosyl-transferring bio-parts and the expensiveness of UDP-arabinose and UDP-xylose, there has been no report on the de novo heterologous biosynthesis of *C*-arabinoside and *C*-xylose in microorganism chassis up to now. Through integration of all genes involved in the flavone *C*-arabinosides and flavone *C*-xylose pathway and introduction of UDP-arabinose and UDP-xylose biosynthesis genes, de novo synthesis of several flavone *C*-arabinosides was preliminarily realized in our engineered *E. coli* strains. Moreover, through high-density fed-batch fermentation, we achieved a high titer of several desired *C*-arabinosides and *C*-xylosides, which proved the feasibility of *E. coli* strains as platform for production of flavone *C*-arabinosides and *C*-xylosides. Unexpectedly, in the fermentation of sCZ113 and sCZ114, the production of isoschaftoside was much lower than schaftoside. This may be due to endogenous dehydratase, yet not identified, preferentially eliminating 2-hydroxyls of 2-OHNar to give a 6-*C*-glucosyl-8-*C*-arabinosyl isomer. This phenomenon is particular because 6-*C*- and 8-*C*- mixture is always observed in the reported work of de novo biosynthesis of *C*-monoglucoside (Vanegas et al. 2018; Sun et al. 2020).

The production of minor product chrysin 6,8-*C*-di-arabinoside was proposed to rise from the promiscuity of tyrosine ammonia lyase (TAL) in pYH55, which recognizes both l-tyrosine and l-phenylalanine as precursors (Li et al. 2019). In addition, significant discrepancy of the productivity between *C*-arabinosides and Api- *C*-xylosides in our constructed strains again supported that UDP-Ara was preferred. This preference of *C*-glycosyltransferases leads to the difference of *C*-glycoside metabolite contents in different rice, which highlighted synthetic biology as more meaningful approach for large-scale manufacturing of rare natural product through the utilization of specific *C*-glycosyltransferases.

*E. coli* does not possess the ability to synthesize UDP-Ara and UDP-Xyl. Introducing an exogenous UDP-Ara and UDP-Xyl biosynthetic pathway to achieve a high production of *C*-glycoside adequately indicated the potential of wider application prospect. Some relevant approaches such as strengthening UDP-Glc supply and replacing Uxs and Uxe from other species will both bring benefits to this pathway. Also, by modification of the C terminal of known CGTs, catalytic pocket mores suitable for UDP-Xyl recognition could be designed, helping engineered strain to reach a higher production of *C*-xylosides. Further study could be focused on downstream products of diglycosides, such as carlinoside, isocarlinoside, lucenin-1 and lucenin-3 if the corresponding flavone 3′-hydroxylase (F3′H) is further incorporated. *E. coli* platform and synthetic biology will become great assist to the development of flavone *C*-arabinosides.

## Supplementary Information

**Additional file1:** **Fig. S1.** An unrooted phylogenetic tree of rice CGTs. **Fig. S2.** LC-MS/MS analyses of minor flavone glycosides present in O. sativa. **Fig. S3.** LC-MS/MS analyses of pathway intermediates in the extracts of sCZ113 and sCZ118. **Fig. S4.** A proposed biosynthetic network of flavone C-xylosides. **Fig. S5.** De novo biosynthesis of vicenin-3 and vicenin-1. **Fig. S6.** NMR spectra of apigenin 6,8-C-di-arabinoside (Api-di-C-Ara). **Fig. S7.** Comparison of 1H NMR spectra of Api-di-C-Ara recorded at different temperature (K). **Fig. S8.** 1H NMR spectra of (A) apigenin 6,8-di-C-xyloside (Api-di-C-Xyl) and (B) chrysin 6,8-di-C-arabinoside (Chr-di-C-Ara) recorded at 353 K. **Fig. S9.** Decomposition of apigenin mono-C-arabinosides. **Fig. S10.** De novo biosynthesis of Api-di-C-Xyl. **Table S1.** Plasmids and strains used in this study. **Table S2.** Primers used in this study.

## Data Availability

All data generated or analyzed during this study are included in this published article (and its supplementary information files).

## Abbreviations

UDP-Glc: UDP-glucose
UDP-Ara: UDP-arabinose
UDP-Xyl: UDP-xylose
Sch: Schaftoside
Isosch: Isoschaftoside
Vic-1: Vicenin-1
Vic-3: Vicenin-3
Phr: Phloretin
Vit: Vitexin
Isovit: Isovitexin
Nar: Naringenin
p-CA: *p*-Coumaric acid
Api-di-C-Ara: Apigenin 6,8-*C*-di-arabinoside
Api-di-C-Xyl: Apigenin 6,8-*C*-di-xyloside
2-OHNar: 2-Hydroxynaringin
