# Supplementary material for: De novo biosynthesis of C-arabinosylated flavones by utilization of indica rice C-glycosyltransferases
Source: Bioresour Bioprocess. 2021 Jun 12;8(1):49. doi: 10.1186/s40643-021-00404-3 (PMC8196924; doi:10.1186/s40643-021-00404-3)
Supplement: Supplementary file 1 — Additional file1: Fig. S1. An unrooted phylogenetic tree of rice CGTs. Fig. S2. LC-MS/MS analyses of minor flavone glycosides present in O. sativa. Fig. S3. LC-MS/MS analyses of pathway intermediates in the extracts of sCZ113 and sCZ118. Fig. S4. A proposed biosynthetic network of flavone C-xylosides. Fig. S5. De novo biosynthesis of vicenin-3 and vicenin-1. Fig. S6. NMR spectra of apigenin 6,8-C-di-arabinoside (Api-di-C-Ara). Fig. S7. Comparison of 1H NMR spectra of Api-di-C-Ara recorded at different temperature (K). Fig. S8. 1H NMR spectra of (A) apigenin 6,8-di-C-xyloside (Api-di-C-Xyl) and (B) chrysin 6,8-di-C-arabinoside (Chr-di-C-Ara) recorded at 353 K. Fig. S9. Decomposition of apigenin mono-C-arabinosides. Fig. S10. De novo biosynthesis of Api-di-C-Xyl. Table S1. Plasmids and strains used in this study. Table S2. Primers used in this study. [file 40643_2021_404_MOESM1_ESM.docx]

**Supplementary Material**

***De novo* biosynthesis of *C*-arabinosylated flavones by utilization of *indica* rice *C*-glycosyltransferases**

Zhuo Chen^1,2#^, Yuwei Sun^1#^, Guangyi Wang^1,2^, Ying Zhang^1,2^, Qian Zhang^1,2^, Yulian Zhang^1,2^, Jianhua Li^1^, Yong Wang^1*^

^1^ CAS-Key Laboratory of Synthetic Biology, CAS Center for Excellence in Molecular Plant Sciences, Institute of Plant Physiology and Ecology, Chinese Academy of Sciences, Shanghai 200032, China. ^2^ University of Chinese Academy of Sciences, Beijing 100039, China. ^*^ Corresponding author. Tel/Fax: 86-21-54924295; Email: [yongwang@cemps.ac.cn](mailto:yongwang@cemps.ac.cn). ^#^ These authors contribute equally to this work

Contents

[Supplementary Figures 3](#_Toc66134270)

[Fig. S1 An unrooted phylogenetic tree of rice CGTs. 3](#_Toc66134271)

[Fig. S2 LC-MS/MS analyses of minor flavone glycosides present in *O. sativa*. 4](#_Toc66134272)

[Fig. S3 LC-MS/MS analyses of pathway intermediates in the extracts of sCZ113 and sCZ118. 5](#_Toc66134273)

[Fig. S4 A proposed biosynthetic network of flavone *C*-xylosides. 7](#_Toc66134274)

[Fig. S5 *De novo* biosynthesis of vicenin-3 and vicenin-1. 8](#_Toc66134275)

[Fig. S6 NMR spectra of apigenin 6,8-*C*-di-arabinoside (Api-di-*C*-Ara). 11](#_Toc66134276)

[Fig. S7 Comparison of ^1^H NMR spectra of Api-di-*C*-Ara recorded at different temperature (K). 12](#_Toc66134277)

[Fig. S8 ^1^H NMR spectra of (A) apigenin 6,8-di-*C*-xyloside (Api-di-*C*-Xyl) and (B) chrysin 6,8-di-*C*-arabinoside (Chr-di-*C*-Ara) recorded at 353 K. 13](#_Toc66134278)

[Fig. S9 Decomposition of apigenin mono-*C*-arabinosides. 14](#_Toc66134279)

[Fig. S10 *De novo* biosynthesis of Api-di-*C*-Xyl. 15](#_Toc66134280)

[Supplementary Tables 16](#_Toc66134281)

[Table S1 Plasmids and strains used in this study. 16](#_Toc66134282)

[Table S2 Primers used in this study. 17](#_Toc66134283)

# Supplementary Figures

## Fig. S1 Unrooted phylogenetic tree of rice CGTs.

Four recently reported dicot CGTs were used as out-group, including NnUGT708N1 (XP_010258947.1), NnUGT708N2 (XP_010265663.2) (Feng et al, 2021), SbCGTa (QLF98861.1), SbCGTb (QLF98862.1) (Wang et al., 2020). The *indica* rice possesses three specific CGTs (OsUGT708A1, OsUGT708A39 and OsUGT708A40, indicated in blue square) belonging to the monocot clade B. Bootstrap values (based on 1000 replications) are indicated at each node.

**
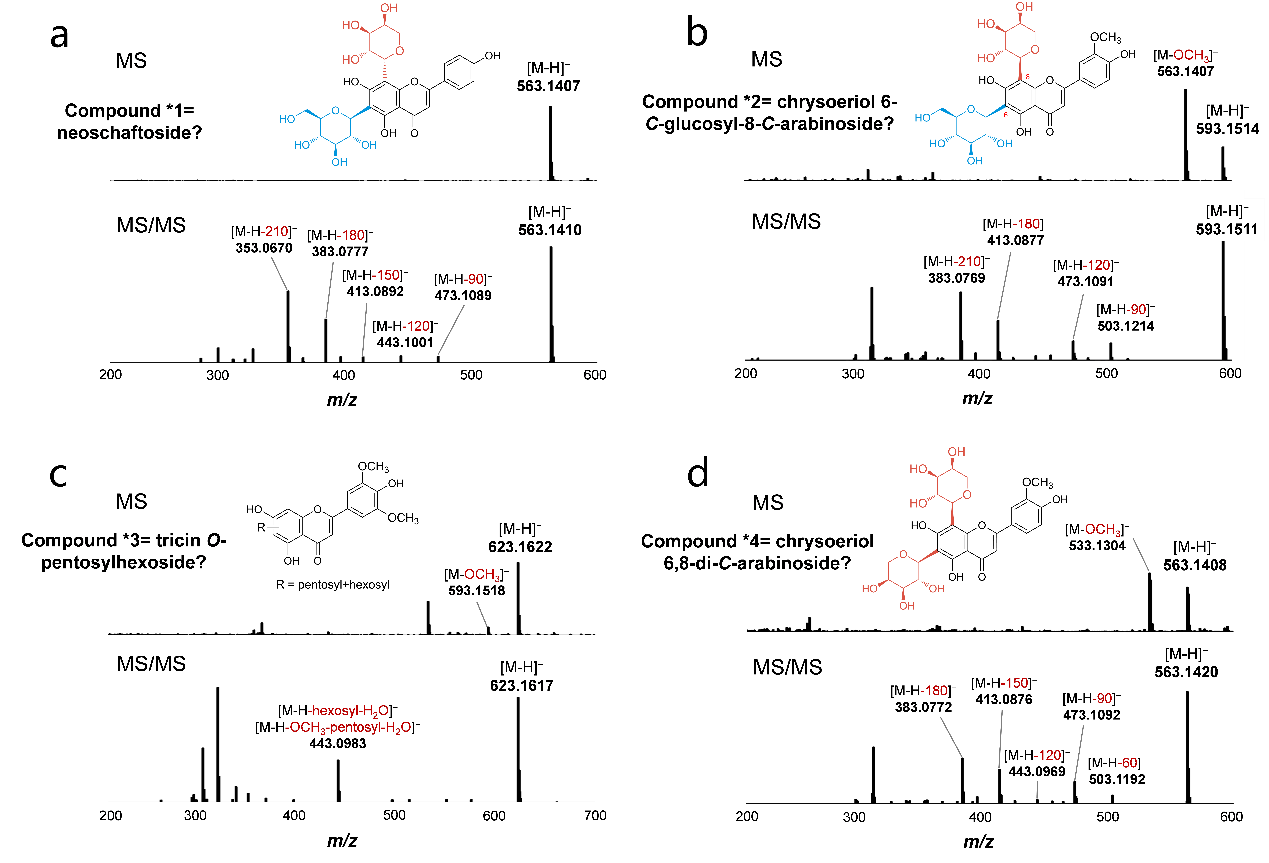
**

## Fig. S2 LC-MS/MS analyses of minor flavone glycosides present in *O. sativa*.

**a** MS and MS/MS fragmentation of compound *1. The *m/z* of compound *1 ([M–H]^–^=563.1) is identical to schaftoside and isoschaftoside. We proposed this compound to be neoschaftoside (Neosch). **b** MS and MS/MS fragmentation of compound *2. Compound *2 showed *m/z* [M–H]^–^=593.2 and [M–H-OCH_3_]^–^=563.1. MS/MS spectrum indicated a hybrid *C*-glycosylation pattern of pentosyl and hexosyl. We proposed this compound to be chrysoeriol 6-*C*-glucosyl-8-*C*-arabinoside or its 6-*C*-arabinosyl-8-*C*-glucoside isomer. **c** Compound *3 with *m/z* [M–H]^–^=623.2 and [M–H-OCH_3_]^–^=593.2 was proposed to be an *O*-pentosylhexoside of tricin, because typical fragmentation of *C*-glycosides was not observed. **d** MS and MS/MS fragmentation of compound *4. We proposed this compound to be di-*C*-arabinoside of chrysoeriol based on MS/MS fragmentation ([M–H-180]^–^, [M–H-150]^–^, [M–H-120]^–^).


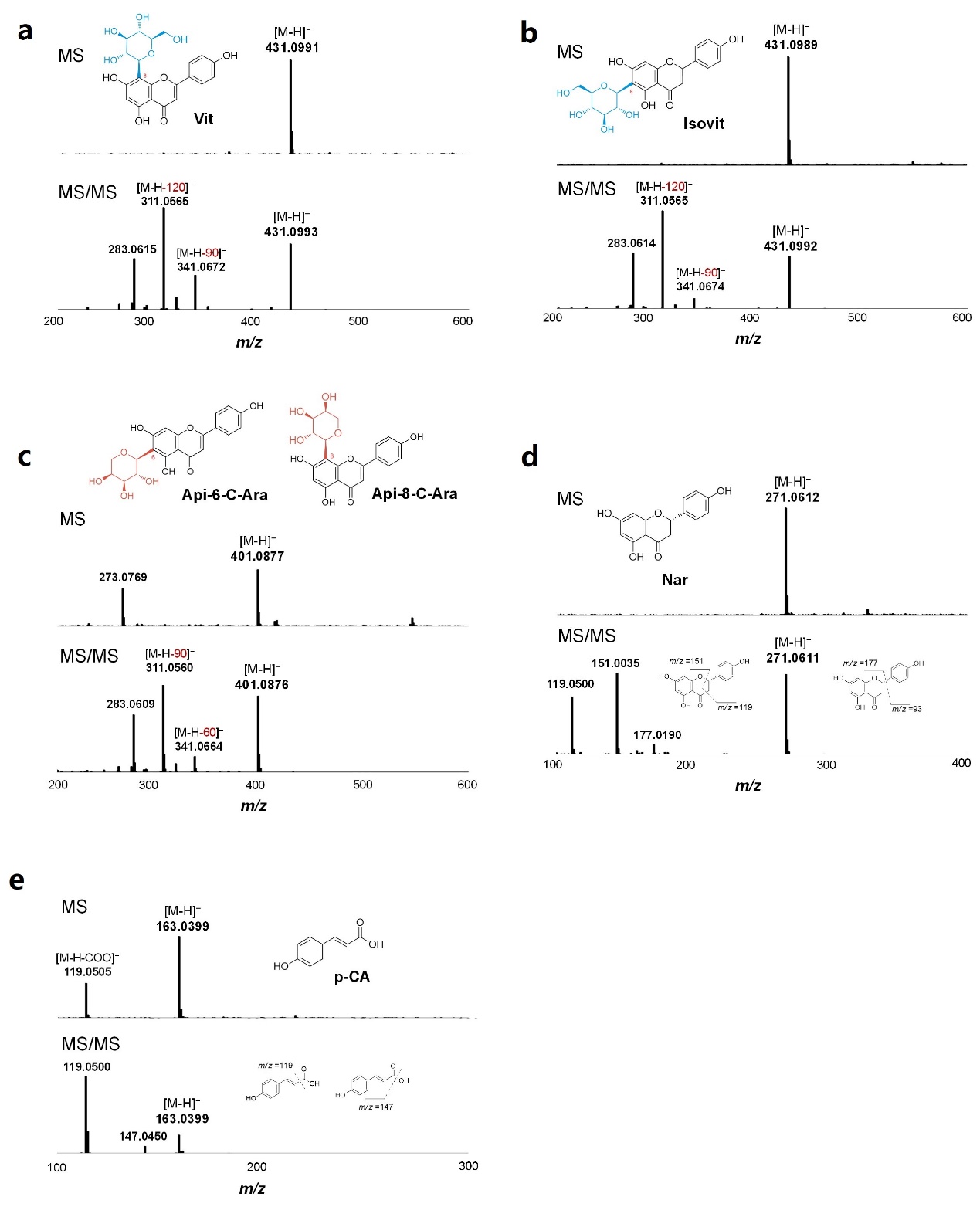


## Fig. S3 LC-MS/MS analyses of pathway intermediates in the extracts of sCZ113 and sCZ118.

**a** MS and MS/MS spectra of vitexin (Vit) observed in the fermentation of sCZ113. **b** MS and MS/MS spectra of isovitexin (Isovit) observed in the fermentation of sCZ113. **c** MS and MS/MS spectra of apigenin 6-*C*-arabinoside (Api 6-*C*-Ara) or apigenin 8-C-arabinoside (Api 8-*C*-Ara) observed in the fermentation of sCZ118. **d** MS and MS/MS spectra of naringenin (Nar) observed in the fermentation of sCZ113 and sCZ118. **e** MS and MS/MS spectra of *p*-coumaric acid (p-CA) observed in the fermentation of sCZ113 and sCZ118.


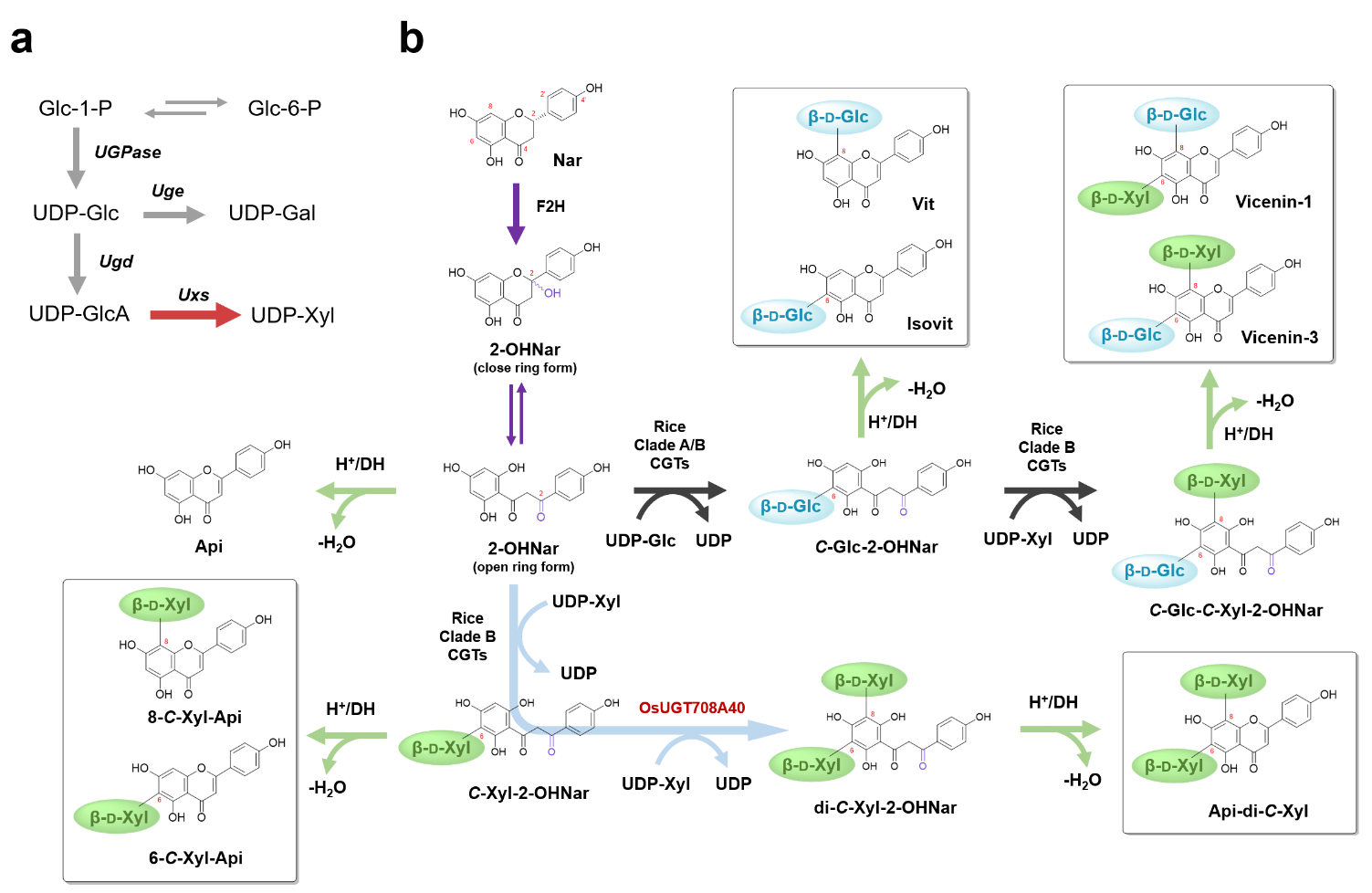


## Fig. S4 A proposed biosynthetic network of flavone *C*-xylosides.

UDP-xylosides (xyl) is generated from glucose-1-phosphate (Glc-1-P) via UDP-glucose (Glc). The purposed *C*-xylosides biosynthesis pathway is highly similar to *C*-arabinosides.

## Fig. S5 *De novo* biosynthesis of vicenin-3 and vicenin-1.

(A) Reconstitution of vicenin-3 and vicenin-1 pathway in *E. coli* chases. pYH55 is assembled for naringenin (Nar) production and pCZ201 harbors cytochrome P450 module for 2-hydroxylnaringenin (2-OHNar) production. (B) Production of sCZ115.

^1^H NMR (500 MHz, DMSO-*d*_6_) spectrum of Api-di-*C*-Ara, at 353 K

^13^C NMR (125 MHz, DMSO-*d*_6_) spectrum of Api-di-*C*-Ara, at 353 K

HSQC spectrum of Api-di-*C*-Ara in DMSO-*d*_6_, at 353 K

HMBC spectrum of Api-di-*C*-Ara in DMSO-*d*_6_, at 353 K

^1^H-^1^H COSY spectrum of sugars of Api-di-*C*-Ara in DMSO-*d*_6_, at 353 K

NOESY spectrum of sugars of Api-di-*C*-Ara in DMSO-*d*_6_, at 353 K

## Fig. S6 NMR spectra of apigenin 6,8-*C*-di-arabinoside (Api-di-*C*-Ara).

**a** ^1^H NMR (500 MHz, DMSO-*d*_6_) spectrum of Api-di-*C*-Ara recorded at 80 ℃ (353 K). **b** ^13^C NMR (125 MHz, DMSO-*d*_6_) spectrum of Api-di-*C*-Ara recorded at 80 ℃ (353 K). **c** HSQC spectrum of Api-di-*C*-Ara in DMSO-*d*_6_. **d** HMBC spectrum of Api-di-*C*-Ara in DMSO-*d*_6_ and key long-range correlations (H→C). **e** ^1^H-^1^H COSY spectrum of sugars of Api-di-*C*-Ara in DMSO-*d*_6_ and key ^1^H-^1^H correlations. **f** 2D-NOESY spectrum of sugars of Api-di-*C*-Ara in DMSO-*d*_6_ and key NOE correlations.

## Fig. S7 Comparison of ^1^H NMR spectra of Api-di-*C*-Ara recorded at different temperature (K).

^1^H NMR (500 MHz, DMSO-*d*_6_) spectrum of Api-di-*C*-Ara recorded at 353 K (80 ℃) showed sharper and more clear signals corresponding to H-2’/H-6’, H-3’/H-5’, H3 and two anomeric protons of arabinose (6-*C*-Ara-H1/8-*C*-Ara-H1), when comparing to those recorded at 323 K (50 ℃) and 293 K (20 ℃).

^1^H NMR (500 MHz, DMSO-*d*_6_) spectrum of Api-di-*C*-Xyl, at 353 K

^1^H NMR (500 MHz, DMSO-*d*_6_) spectrum of Chr-di-*C*-Ara, at 353 K

## Fig. S8 ^1^H NMR spectra of a apigenin 6,8-di-*C*-xyloside (Api-di-*C*-Xyl) and b chrysin 6,8-di-*C*-arabinoside (Chr-di-*C*-Ara) recorded at 353 K.

## Fig. S9 Decomposition of apigenin mono-*C*-arabinosides.

HPLC analyses of sCZ113 extracts (re-dissolved in 50% methanol) right after extraction (0 h), incubated at room temperature for 24 h and 48 h. Gradual decrease of the peak of apigenin 6-*C*-arabinoside (or apigenin 8-*C*-arabinoside) was observed. UV absorbance at 340 nm was monitored.

## Fig. S10 *De novo* biosynthesis of Api-di-*C*-Xyl.

**a** Reconstitution of Api-di-C-Xyl pathway in E. coli chases. pYH55 is assembled for naringenin (Nar) production and pCZ201 harbors cytochrome P450 module for 2-hydroxylnaringenin (2-OHNar) production. **b** Production of sCZ119.

# Supplementary Tables

## Table S1 Plasmids and strains used in this study.

| ID | Description | Source/references |  | ID | Description | Source/references |
| --- | --- | --- | --- | --- | --- | --- |
| **Plasmids** |  |  |  | **Strains** |  |  |
| pYH055 | pCDF-T7-4CL-T7-PAL-T7-CHS-T7-CHI | (Li et al, 2019) |  | *E. coli* BL21(DE3) | *E. coli* chasis for pathway engineering | NEB |
| pCZ201 | pET-Duet-T7-2B1-tr29_Syn_ZmCYP93G5-T7-AtCPR2 | (Sun et al, 2020) |  | sCZ110 | BL21(DE3): pYH055+pCZ201+pET28a | This work |
| pCZ86 | pET-28a-T7-PhUGT708A43 | (Sun et al, 2020) |  | sCZ112 | BL21(DE3): pYH055+pCZ201 | (Sun et al, 2020) |
| pCZ162 | pET-28a-T7-OsUGT708A2 | (Sun et al, 2020) |  | sCZ113 | BL21(DE3): pYH055+pCZ201+pCZ193-1 | This work |
| pCZ163 | pET-28a-T7-OsUGT708A1 | (Sun et al, 2020) |  | sCZ114 | BL21(DE3): pYH055+pCZ201+pCZ193-2 | This work |
| pCZ164 | pET-28a-T7-OsUGT708A39 | (Sun et al, 2020) |  | sCZ118 | BL21(DE3): pYH055+pCZ201+pCZ194 | This work |
| pCZ165 | pET-28a-T7-OsUGT708A40 | (Sun et al, 2020) |  | sCZ119 | BL21(DE3): pYH055+pCZ201+pCZ195 | This work |
| pCZ191 | pET-28a-T7-PhUGT708A43-OsUGT708A1 | This work |  |  |  |  |
| pCZ192-1 | pET-28a-T7-PhUGT708A43-OsUGT708A1-SmUxs1 | This work |  |  |  |  |
| pCZ192-2 | pET-28a-T7-PhUGT708A43-OsUGT708A1-SmUxs2 | This work |  |  |  |  |
| pCZ193-1 | pET-28a-T7-PhUGT708A43-OsUGT708A1-SmUxs1-SmUxe | This work |  |  |  |  |
| pCZ193-2 | pET-28a-T7-PhUGT708A43-OsUGT708A1-SmUxs2-SmUxe | This work |  |  |  |  |
| pCZ194 | pET-28a-T7-OsUGT708A40-SmUxs1-SmUxe | This work |  |  |  |  |
| pCZ195 | pET-28a-T7-OsUGT708A40-SmUxs1 | This work |  |  |  |  |

## Table S2 Primers used in this study.

| Name | Primers | Sequence (5’→3’) |
| --- | --- | --- |
| pCZ191 | pCZ191-F | ATCTTGCAGTGTTGGACTAGTTTGTTTAACTTTAAGAAGGAGATATACCATG |
|  | pCZ191-R | gtggtggtggtgctcgagtAAGCTTgcgggatccgccgcTTAAGTGGCCTTGAGCTTTT |
| pCZ192-1 | pCZ192-1-F | GAAAAGCTCAAGGCCACTTAAgcggcaataattttgtttaactttaagaa |
|  | pCZ192-1-R | gtggtggtggtgctcgagtAAGCTTgcgggatccTCAGACCAGCTCCGCACT |
| pCZ192-2 | pCZ192-2-F | GAAAAGCTCAAGGCCACTTAAgcggcaataattttgtttaactttaagaa |
|  | pCZ192-2-R | tggtggtggtggtgctcgagtAAGCTTgcgggatccTCACAGATCCTGACTGACGAC |
| pCZ193-1 | pCZ193-1-F | AAAGTGCGGAGCTGGTCTGAgcggcaataattttgtttaactttaag |
|  | pCZ193-1-R | tggtggtgctcgagtGCGGCagggatcctcatgctagcCGcTCATGACCGGACCTCCAG |
| pCZ193-2 | pCZ193-2-F | TCGTCAGTCAGGATCTGTGAgcggcaataattttgtttaactttaag |
|  | pCZ193-2-R | tggtggtgctcgagtGCGGCagggatcctcatgctagcCGcTCATGACCGGACCTCCAG |
| pCZ194 | pCZ194-F | GCAAAGCTCAAGGCTGCTTAgcggcaataattttgtttaactttaagaag |
|  | pCZ194-R | tggtggtgctcgagtGCGGCagggatcctcatgctagcCGcTCATGACCGGACCTCCAG |
| pCZ195 | pCZ195-F | AAAGTGCGGAGCTGGTCTGAactcgagcaccaccac |
|  | pCZ195-R | TCAGACCAGCTCCGCAC |

**Reference**

Feng C, Li S, Taguchi G, Wu Q, Yin D, Gu Z, Wu J, Xu W, Liu C, Wang L (2021) Enzymatic basis for stepwise *C*-glycosylation in the formation of flavonoid di-*C*-glycosides in sacred lotus (*Nelumbo nucifera* Gaertn.). Plant J. <https://doi.org/10.1111/tpj.15168>

Li J, Tian C, Xia Y, Mutanda I, Wang K, Wang Y (2019) Production of plant-specific flavones baicalein and scutellarein in an engineered *E. coli* from available phenylalanine and tyrosine. Metab Eng 52**:**124-133. <https://doi.org/10.1016/j.ymben.2018.11.008>

Sun Y, Chen Z, Yang J, Mutanda I, Li S, Zhang Q, Zhang Y, Zhang Y, Wang Y (2020) Pathway-specific enzymes from bamboo and crop leaves biosynthesize anti-nociceptive *C*-glycosylated flavones. Commun Biol 3**:**110-110. <https://doi.org/10.1038/s42003-020-0834-3>

Wang Z, Gao H, Wang S, Zhang M, Chen K, Zhang Y, Wang H, Han B, Xu L, Song T, Yun C, Qiao X, Ye M (2020) Dissection of the general two-step di-*C*-glycosylation pathway for the biosynthesis of (iso)schaftosides in higher plants. Proc Natl Acad Sci USA. Doi:10.1073/pnas.2012745117. <https://doi.org/10.1073/pnas.2012745117>
